# Ammonium and nitric oxide condition the establishment of *Arabidopsis* L*er*/Kas-2 immune-related hybrid incompatibility

**DOI:** 10.1007/s00425-022-03990-4

**Published:** 2022-09-10

**Authors:** Kostadin Evgeniev Atanasov, Lucía C. Díaz-Narváez, Rubén Alcázar

**Affiliations:** Department of Biology, Healthcare and Environment, Section of Plant Physiology, Faculty of Pharmacy and Food Sciences, Universitat de Barcelona, 08028 Barcelona, Spain

**Keywords:** Autoimmunity, Defense, Nitrogen nutrition, NPR1, Reactive nitrogen species, Reactive oxygen species

## Abstract

**Main Conclusion:**

**High ammonium suppresses hybrid incompatibility between Ler and Kas-2 accessions through lowering nitric oxide levels and nitrate reductase activity required for autoimmunity.**

**Abstract:**

The immune-related hybrid incompatibility (HI) between Landsberg *erecta* (L*er*) and Kashmir-2 (Kas-2) accessions is due to a deleterious genetic interaction between the *RPP1* (*RECOGNITION OF PERONOSPORA PARASITICA1*)-like L*er* locus and Kas-2 alleles of the receptor-like kinase *SRF3* (*STRUBBELIG RECEPTOR FAMILY 3*). The genetic incompatibility is temperature-dependent and leads to constitutive activation of the salicylic acid (SA) pathway, dwarfism and cell death at 14–16 °C. Here we investigated the effect of nutrition on the occurrence of L*er*/Kas-2 HI and found that high ammonium suppresses L*er*/Kas-2 incompatible phenotypes independently of the ammonium/nitrate ratio. Ammonium feeding leads to compromised disease resistance to *Pseudomonas syringae* pv. *tomato* DC3000, lower total SA, nitric oxide and nitrate reductase activity in L*er*/Kas-2 incompatible hybrids. In addition, we find that L*er*/Kas-2 incompatibility is dependent on NPR1 (NONEXPRESSER OF PR GENES 1) and nitric oxide production. Overall, this work highlights the effect of nutrition on the expression of incompatible phenotypes independently of temperature.

**Supplementary Information:**

The online version contains supplementary material available at 10.1007/s00425-022-03990-4.

## Introduction

*Arabidopsis thaliana* (*Arabidopsis*) is a naturalized species found in different environments which helps at the study of evolutionary adaptation to different habitats (Koornneef et al. 2004). Exploring *Arabidopsis* within-species variation revealed the occurrence of deleterious genetic interactions involving disease *Resistance* genes (*R*) from different parental lineages leading to constitutive activation of defense responses or autoimmunity (Bomblies et al. 2007; Alcázar et al. 2009; Smith et al. 2011; Chae et al. 2014; Vaid and Laitinen 2019). This syndrome is referred to as immune-related hybrid incompatibility (HI). Due to the high costs on growth and reproduction, immune-related HIs are proposed to influence gene flow between populations and to be a potential basis for plant speciation (Bomblies and Weigel 2007; Schumer et al. 2015).

The first temperature-dependent HI reported in *Arabidopsis* was described between the accessions Umkirch-1 (Uk-1) and Umkirch-3 (Uk-3), originally collected from the same local population (Bomblies et al. 2007). Uk-1/Uk-3 F1 hybrids exhibit dwarfism and leaf necrosis at low temperature (14 °C). Uk-1/Uk-3 HI involves a two-way epistatic interaction between two loci, namely, *DANGEROUS MIX 1 *(*DM1*) of Uk-3 and *DANGEROUS MIX 2 *(*DM2*) of Uk-1. *DM1* maps to *SUPPRESSOR OF SALICYLIC ACID INSENSITIVITY OF NPR1* 4 (*SSI4*). The *DM2* locus maps to the *RPP1* (*RECOGNITION OF PERONOSPORA PARASITICA 1*) locus containing seven TIR–NB–LRR (toll/interleukin-1 receptor–nucleotide binding leucine rich repeat) genes (Bomblies et al. 2007; Chae et al. 2014). Through a systematic analysis of several F1 populations, other incompatible genetic interactions have been identified that also involve the *DM2* locus. This locus might be considered a ‘hot spot’ of immune-related HIs (Chae et al. 2014).

We previously reported a genetic interaction between the *RPP1*-like locus of Landsberg *erecta* (L*er*) (QTL3 or DM2 L*er*) and the Kashmir (Kas-2) allele of the receptor-like kinase *Strubbelig-Receptor Family 3* (*SRF3*) (QTL4) triggering immune-related HI (Alcázar et al. 2009). The L*er*/Kas-2 HI was reconstituted in a near isogenic line (NIL) containing an *RPP1*-like L*er* introgression in the Kas-2 genetic background (L*er*/Kas-2 NIL). L*er*/Kas-2 HI is temperature-dependent and associates with constitutive activation of defenses at 14–16 °C, which are suppressed at higher temperature (20–22 °C). Cell death, dwarfism, and constitutive activation of salicylic acid (SA) pathway are hallmarks of L*er*/Kas-2 HI (Alcázar et al. 2009). The incompatible *RPP1*-like L*er* haplotype was found at a frequency of 30% in a local population of *Arabidopsis* in Gorzów Wielkopolski (Poland), where the Landsberg accession was originally collected in 1939 (Alcázar et al. 2014). More recently, EMS and CRISPR/Cas9-directed mutagenesis revealed that L*er*/Kas-2 HI is fully suppressed by *RPP1*-like *R8* loss-of-function mutations (Atanasov et al. 2018). Here we report that, in addition to temperature, nutrient availability is another important factor influencing the expression of L*er*/Kas-2 immune-related HI. After carbon, ammonium (NH_4_^+^), and nitrate (NO_3_^−^) are the most important nutrients for plant growth. Their deficiency reduces crop production, whereas their overaccumulation triggers toxicity and negative effects on plant growth and yield (Li et al. 2014). NH_4_^+^ is quickly oxidized into NO_3_^−^ by soil microbiota. The levels of NH_4_^+^ in forest soils range between 0.4 and 4 mM, whereas in agricultural lands ammonium levels raise up to 20 mM or even higher concentrations under low oxidizing conditions (Britto and Kronzucker 2002; Li et al. 2014). Soil pollution by ammonium is mainly caused by human activity, such as over-fertilization, intensive cattle, industrial activities, and organic waste. While nitrogen input increases plant growth and yield, it can also be used by pathogens to grow, thus establishing a competition for nutrients between plants and pathogens (Tavernier et al. 2007). Indeed, the availability of nutrients is essential for pathogenesis (Solomon et al. 2003). As such, plants reallocate nutrients from the apoplast to restrict their availability for pathogenic growth at infection sites (Cao et al. 2015; Schwachtje et al. 2018). The effect of nitrogen nutrition on defense responses cannot be neglected (Mur et al. 2017). In this work, we investigated the effect of nitrogen nutrition on the modulation of L*er*/Kas-2 immune-related HI. We further analyzed the contribution of nitric oxide (NO) and reactive oxygen species (ROS) to autoimmune phenotypes, and provide genetic evidence for the requirement of *NONEXPRESSOR OF PATHOGENESIS-RELATED GENES1* (*NPR1*) to L*er*/Kas-2 incompatibility.

## Materials and methods

### Plant materials

The L*er* and Kas-2 accessions, as well as the L*er*/Kas-2 near isogenic line (NIL) were previously described (Alcázar et al. 2009, 2010). The *npr1-1* (Col-0) loss-of-function mutant (CS3726) (Cao et al. 1997) was obtained from the Nottingham *Arabidopsis* Stock Centre (http://arabidopsis.info).

### Hydroponic system

A hydroponic system was established to test the effect of nutrition on L*er*/Kas-2 HI. Syringe cylinders (Becton Dickinson) were filled with 10 ml glass beads (2.8–3.4 mm diameter). The upper surface of the cylinder was covered with sand Fontainebleau (VWR, Darmstadt, Germany), and the resulting bed was washed 10 times with sterile ddH_2_O before filling with the appropriate nutrient solution. The cylinders were placed into a hydroponic system (http://www.araponics.com). Plants were grown under 16 h light/8 h dark cycles at the indicated temperature, 70% relative humidity and 160 μmol photons m^−2^ s^−1^ light intensity. The nutritional media was replaced every 2 days.

### Treatments with cPTIO and DMTU

The nitric oxide (NO) scavenger cPTIO [2-(4-carboxyphenyl)-4,4,5,5-tetramethylimidazoline-1-oxyl-3-oxide] and the hydrogen peroxide (H_2_O_2_) scavenger DMTU (1,3-dimethyl-2-thiourea) were purchased from Merck. Five-day-old seedlings grown in vitro on low-ammonium MS at 14–16 °C were transferred to new MS media containing 0.1 mM cPTIO (Gao et al. 2013) or 0.1 mM DMTU (Fraudentali et al. 2021) and incubated 48 h further before harvesting in liquid nitrogen for total RNA extraction.

### Quantitative real-time PCR gene expression analysis (qRT-PCR)

Total RNA was extracted using TRI Reagent (Merck) according to manufacturer’s instructions. Two micrograms of RNA incubated with DNase I (Invitrogen) and the cDNA synthesized using Superscript IV (Invitrogen). qRT-PCR was performed using SYBR Green Master Mix kit (Bio-Rad) on a Roche Light Cycler 480 II device, using the following PCR conditions: 95 °C for 15 s; 60 °C for 15 s and 68 °C for 1 min. Standard curves were performed for quantitation. Primer sequences used for *PR1* and *GST1* gene expression analyses were previously reported (Alcázar et al. 2009). *ACTIN2* (*At3g18780*) was used as housekeeping gene for qRT-PCR analyses (Alcázar et al. 2009).

### Hydrogen peroxide quantitation

Hydrogen peroxide was quantified according to Velikova et al. (2000). Briefly, 10 mg of deep-frozen and homogenized plant material was transferred to 1.5 ml tubes and mixed with 0.5 ml fresh 0.1% (w/v) trichloroacetic acid (TCA). Samples were vortexed for 30 s, incubated on ice for 15 min and then centrifuged at 15,000g for 15 min at 4 °C. The supernatant was then transferred to a new tube and mixed with 0.5 ml 10 mM potassium phosphate buffer (pH = 7.0). The reaction was started by addition of 1 ml potassium iodide (1M) and the absorbance determined at 390 nm.

### Nitric oxide quantitation

Nitric oxide levels were determined according to Zhou et al. (2005) by indirect quantification of nitrite-derived NO. Briefly, deep-frozen homogenized plant material (20 mg) was extracted with 0.5 ml 50 mM acetic acid and 4% (w/v) zinc acetate (pH = 3.6). Samples were incubated on ice for 15 min and then centrifuged at 15,000g for 15 min at 4 °C. The supernatant was collected to a new tube and the pellet extracted once more as described before. Both supernatants were collected, activated with charcoal and centrifuged at 15,000g for 15 min at 4 °C. One volume of Griess reagent (Sigma-Aldrich) was added to start the reaction. After 30 min of incubation at room temperature, the absorbance was determined at 540 nm.

### Nitrate reductase activity measurements

Nitrate reductase (NR) activity was determined according to the method described by Park et al. (2011). Deep-frozen plant material was homogenized in extraction buffer (250 mM Tris–HCl (pH 8.0), 1 mM EDTA, 1 μM Na_2_MoO_4_, 5 μM flavin adenine dinucleotide, 3 mM dithiothreitol, 1% BSA, 12 mM *β*-mercaptoethanol and 250 μM PMSF) and centrifuged at 15,000g for 15 min at 4 °C. The supernatant was collected and mixed with the reaction buffer (40 mM NaNO_3_, 80 mM Na_2_HPO_4_, 20 mM NaH_2_PO_4_ (pH 7.5) and 0.2 mM NADH). The reaction was halted by the addition of 1% sulphanilamide and 0.05% *N*-(1-napthyl) ethylenediamine hydrochloride after further incubation for 2 h at room temperature. The concentration of nitrite was determined by measuring the absorbance at 540 nm.

### Superoxide dismutase (SOD) activity

Superoxide dismutase activity was determined using the SOD Assay Kit (19160-1KT-F, Sigma) following manufacturer’s instructions. Leaves from 5-week-old plants were grinded in liquid nitrogen and homogenized in ice-cold 50 mM potassium hydrogen phosphate buffer (pH = 7.8) containing 0.1 mM ascorbic acid, 1 mM PMSF, 1 mM EDTA, 0.05% Triton X-100 and 0.05% *β*-mercaptoethanol (Van Camp et al. 1994). The crude extract was clarified by centrifugation at 15,000g 15 min, 4 °C, and total protein quantified by the Bradford method. SOD activity was expressed as percentage of the enzymatic inhibition rate normalized to the total protein amount.

### Catalase activity

Leaves from 5-week-old plants were grinded in liquid nitrogen and homogenized in ice-cold 50 mM potassium phosphate buffer (pH = 7.0), 0.5 mM ascorbic acid, 0.1 mM EDTA, 1 mM PMSF and 0.05% Triton X-100. The crude extract was clarified by centrifugation at 15,000g for 15 min, 4 °C. Catalase activity was determined according to (Senthilkumar et al. 2021) and expressed as catalase enzymatic activity (U) normalized to the total protein amount.

### Salicylic acid quantitation

SA quantitation was performed according to Defraia et al. (2008). Samples (200 mg) were homogenized in liquid nitrogen, resuspended in 250 µl 100 mM sodium acetate (pH = 5.5) and incubated on ice for 30 min. Samples were then centrifuged at 15,000g for 15 min at 4 °C. The supernatant (200 µl) was treated with 2U *β*-glucosidase and incubated at 37 °C for 1.5 h. Samples were then frozen 3 h for enzymatic deactivation. Bacteria incubation was performed by adding 50 µl of *Acinetobacter ADPWH_lux* cell suspension (OD_600_ = 0.4) to 200 µl of the plant extract and incubated at 37 °C for 1 h. A negative control (L*er NahG*) (Alcázar et al. 2009) was used for background subtraction.

### *Pseudomonas syringae* pv. *tomato DC3000* (*Pst DC3000*) inoculation

Inoculations with *Pseudomonas syringae pv*. *tomato* DC3000 (*Pst* DC3000) were performed in 5-week-old plants by spray-inoculation with a bacterial suspension of 1 × 10^8^ cfu/ml in 10 mM MgCl_2_ with 0.04% (v/v) Silwet L-77 (Lehle Seeds, Round Rock, TX, USA). In planta bacterial titers were determined 3 days after inoculation as described (Alcázar et al. 2010).

### Trypan blue staining

Plant cell death was visualized by staining with lactophenol trypan blue (Alcázar et al. 2009). Samples were mounted in 60% glycerol, observed under light microscope and images captured in a Moticam 5.0 MP camera.

### CAPS genotyping

F2 plants from the cross *npr1-1* × L*er*/Kas-2 NIL were genotyped with CAPS markers flanking the incompatible loci on QTL3, QTL4 and QTL5 as reported in Alcázar et al. (2010). The Col-0 allele carrying the *npr1-1* mutation was genotyped with the SSLP markers F11P17 (Fwd: 5'CGCAATCGATTTTATTTAAATCC; Rev: 5'TTTCAGTTTGATGATTTATTCGC) and F5I14 (Fwd: 5'CTGCCTGAAATTGTCGAAAC; Rev: 5'GGCATCACAGTTCTGATTCC). The *npr1-1* mutation from the quadruple homozygous L*er*/Kas-2 *npr1-1* (QTL3: L*er*/L*er*, QTL4: Kas-2/Kas-2; QTL5:Kas-2/Kas-2; *npr1-1*/*npr1-1*) was sequenced by PCR amplification using the primers: NPR1_Fwd: 5'CGCTACCGATAACACCGACT and NPR1_Rev: 5'TCGTTTCTCAGCAGTGTCGT.

## Results

### High ammonium suppresses L*er*/Kas-2 hybrid incompatibility

**Fig. 1:**
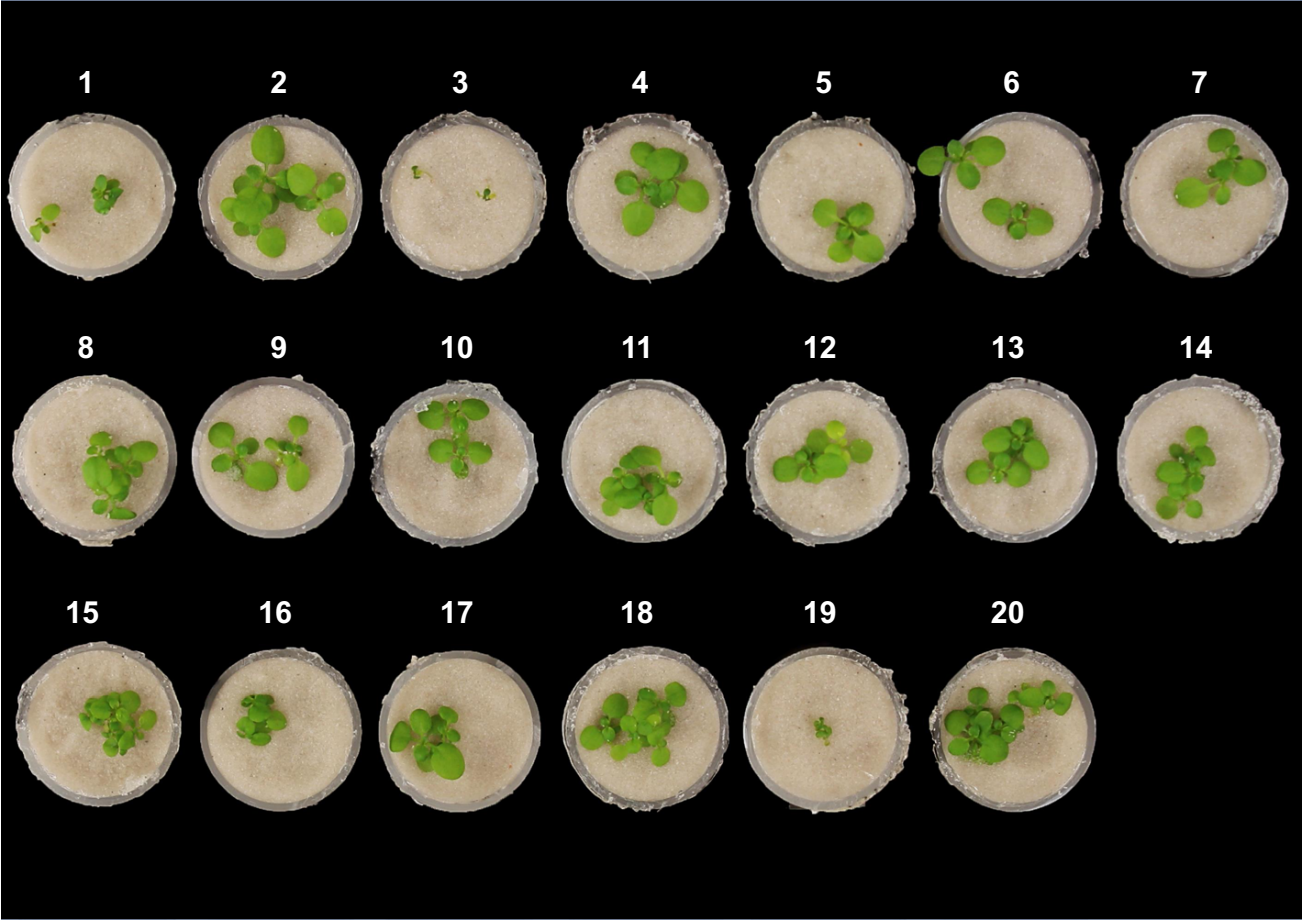
Phenotype of 3-week-old L*er*/Kas-2 NIL plants grown hydroponically at 14–16 °C under different nutrient conditions. The concentration of each salt in the MS formulation was reduced to the corresponding HS concentration: (1) 1 mM NH_4_NO_3_, (2) 2 mM KNO_3_, (3) 0 mM CaCl_2_, (4) 0 mM KI, (5) 500 µM MgSO_4_, (6) 1 mM KH_2_PO_4_, (7) 2 mM Ca(NO_3_)_2_, (8) 19 µM Fe-EDDHA, (9) 9 µM H_3_BO_3_, (10) 15 µM MnSO_4_, (11) 5 µM ZnSO_4_, (12) 5 µM CuSO_4_, (13) 0.3 µM CoCl_2_, (14) 0.9 µM Na_2_MoO_3_, (15) 0.5 × HS + Gamborg’s B6 vitamins, (16) 0.5 × HS, (17) 0.5 × MS + Gamborg’s B6 vitamins, (18) 0.5 × MS, (19) H_2_O, (20) 0.5 × MS-MES plus Gamborg’s B6 Vitamins

We previously reported that L*er/*Kas-2 HI was evidenced in L*er*/Kas-2 NIL (NIL) plants grown on soil at low temperature (14–16 °C). However, incompatibility was suppressed in L*er*/Kas-2 NIL plants grown in vitro (0.5 × Murashige and Skoog basal medium, MS) at low temperature (Alcázar et al. 2009). During the course of our experiments, we found that hydroponic growth of L*er*/Kas-2 NIL plants in 0.5 × Hoagland’s nutrient solution (HS) reconstituted HI phenotypes at 14–16 °C (Fig. 1). In contrast, the use of a nutrient solution based on 0.5 × MS suppressed hybrid incompatibility (Fig. 1). We hypothesized that MS media composition rather than high humidity or aseptic growth might be causal for the suppression of L*er*/Kas-2 incompatible phenotypes. The MS basal medium is formulated for plant in vitro culture and contains higher concentrations for most nutrients compared to HS. To identify which nutrient(s) in MS suppressed the incompatible phenotypes of L*er*/Kas-2 NIL at 14–16 °C, we tested each salt in the MS formulation by reducing its concentration to HS levels (Fig. 1; Table S1). Lowering NH_4_NO_3_ concentration from 10.31 mM (MS) to 1 mM (HS) was sufficient to reconstitute dwarfism of L*er*/Kas-2 NIL plants grown in a hydroponic culture at 14–16 °C. In contrast, lowering KNO_3_ concentration from 9.40 mM (MS concentration) to 2 mM (HS concentration) did not reconstitute L*er*/Kas-2 HI (Fig. 1).

**Fig. 2:**
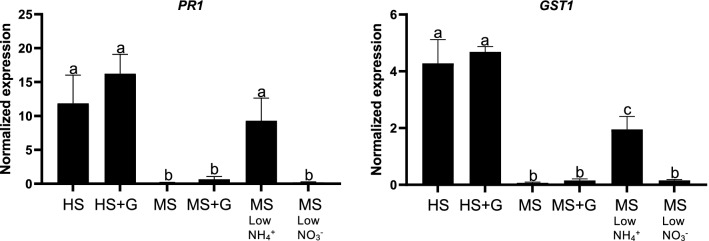
Quantitative gene expression analyses of *PR1* and *GST1* in 3-week-old L*er*/Kas-2 NIL plants grown hydroponically at low temperature (14–16 °C) in 0.5 × Hoagland’s solution (HS), 0.5 × Hoagland’s solution supplemented with Gamborg’s vitamins (HS + G), 0.5 × Murashige and Skoog solution (MS), 0.5 × Murashige and Skoog solution supplemented with Gamborg’s vitamins, low ammonium MS (0.5 × MS containing 1 mM NH_4_NO_3_), and low nitrate MS × (0.5 × MS containing 2 mM KNO_3_). qRT-PCR analyses were performed on at least three biological replicates with three technical replicates each. Letters indicate values that are significantly different according to Tukey’s HSD test at *P* < 0.05

**Fig. 3:**
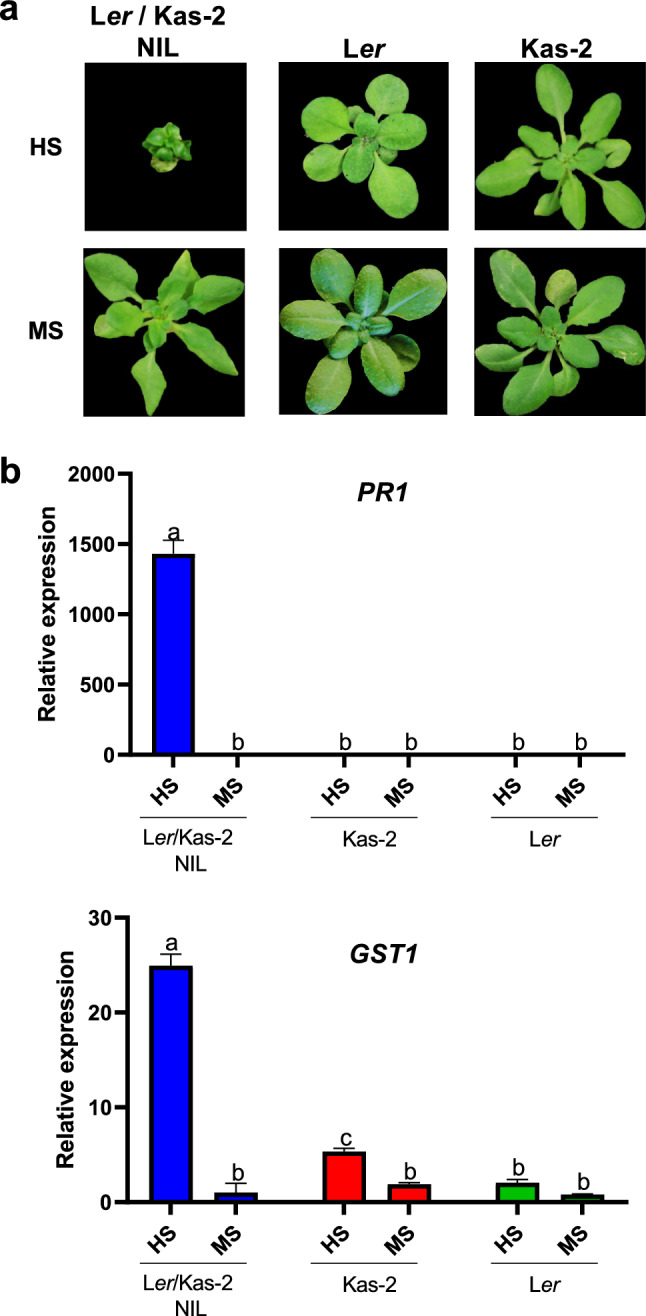
**a** Phenotype of 5-week-old L*er*, Kas-2 and L*er*/Kas-2 NIL plants grown on soil at 14–16 °C and irrigated with Hoagland’s solution (HS) or Murashige and Skook (MS) solution. **b** qRT-PCR gene expression analyses of *PR1* and *GST1* in L*er*, Kas-2 and L*er*/Kas-2 NIL plants grown as indicated in **a**

L*er*/Kas-2 HI leads to constitutive SA-pathway activation and upregulation of the oxidative stress marker gene *GST1* (*GLUTATHIONE-S-TRANSFERASE1*) (Alcázar et al. 2009). Consistent with the suppression of incompatible phenotypes, L*er*/Kas-2 NIL plants grown on MS (supplemented or not with Gamborg’s B6 vitamins) or low nitrate MS (2 mM KNO_3_) showed significantly lower *PR1* and *GST1* expression than L*er*/Kas-2 NIL plants grown on HS, HS (supplemented or not with Gamborg’s B6 vitamins) and low ammonium MS (1 mM NH_4_NO_3_) (Fig. 2). The suppressive effect of high ammonium on dwarfism, cell death, *PR1* and *GST1* expression was also observed in L*er*/Kas-2 plants grown on soil and irrigated with MS compared to HS (Fig. 3). We concluded that irrigation or hydroponic growth of L*er*/Kas-2 incompatible hybrids under high ammonium suppresses incompatibility.

### Effect of the NH_4_^+^/NO_3_^−^ ratio in the suppression of L***er***/Kas-2 hybrid incompatibility

**Fig. 4:**
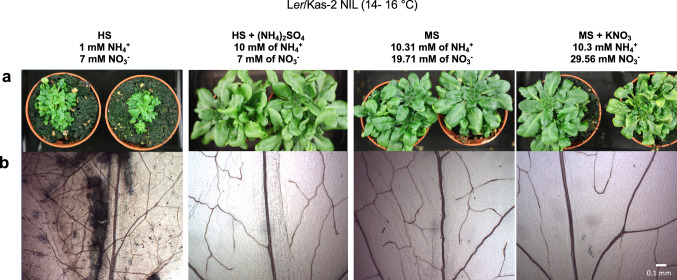
**a** Phenotype of 5-week-old L*er*/Kas-2 NIL plants grown on soil at 14–16 °C and irrigated with different ammonium:nitrate ratios as indicated in the HS, HS + (NH_4_)_2_ SO_4_, MS and MS + KNO_3_ treatments. **b** Trypan blue staining of leaves from L*er*/Kas-2 NIL plants treated as described in **a**

To further study whether higher ammonium concentration or higher NH_4_^+^/NO_3_^−^ ratio associated with the suppression of incompatible phenotypes, L*er*/Kas-2 NIL plants were irrigated with nutrient solutions containing different NH_4_^+^/NO_3_^−^ ratios: HS (1 mM NH_4_^+^/7 mM NO_3_^−^), HS supplemented with ammonium sulfate (NH_4_)_2_SO_4_ (10 mM NH_4_^+^/7 mM NO_3_^−^), MS (10.31 mM NH_4_^+^/19.71 mM NO_3_^−^) and MS supplemented with potassium nitrate KNO_3_ (10.3 mM NH_4_^+^/29.56 mM NO_3_^−^) (Fig. 4). Irrigation with 10 mM and higher ammonium concentrations suppressed L*er*/Kas-2 NIL dwarfism and cell death at 14–16 °C regardless of the NH_4_^+^/NO_3_^−^ ratio. In contrast, increases in nitrate concentration did not counteract the suppressive effect of high NH_4_^+^ on L*er*/Kas-2 dwarfism and cell death. We concluded that high ammonium is sufficient to suppress L*er*/Kas-2 HI independently of NO_3_^−^ levels.

### Analysis of SA levels in L*er*/Kas-2 NIL and parental lines irrigated with HS or MS

**Fig. 5:**
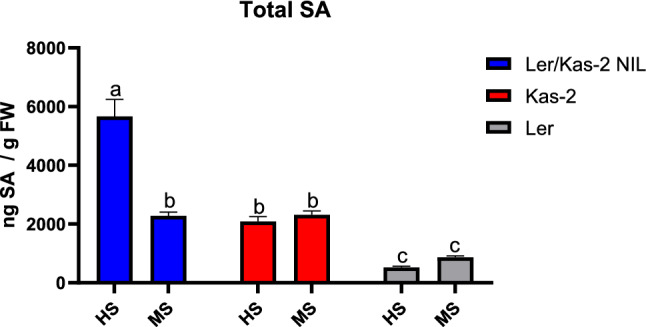
Total salicylic acid (SA) levels in 5-week-old L*er*/Kas-2 NIL, L*er* and Kas-2 plants grown at 14–16 °C. Values represent the average ± SD from five biological replicates. Letters indicate values that are significantly different according to Tukey’s HSD test at *P* < 0.05

L*er*/Kas-2 HI is SA-dependent and L*er*/Kas-2 NIL plants accumulate higher SA levels than parental lines (L*er* and Kas-2) at 14–16 °C (Alcázar et al. 2009). To investigate the effect of nutrition on SA content, total SA was determined in L*er*/Kas-2 NIL, L*er* and Kas-2 plants grown at 14–16 °C and irrigated with MS or HS (Fig. 5). Consistent with the occurrence of incompatible phenotypes, L*er*/Kas-2 NIL plants irrigated with HS accumulated significantly higher SA than L*er* or Kas-2. In contrast, L*er*/Kas-2 NIL irrigated with MS exhibited similar SA levels compared to the near isogenic Kas-2 background. Irrigation with MS or HS did not lead to significant changes in basal SA in the parental lines (Fig. 5). Overall, suppression of L*er*/Kas-2 HI by MS associated with compromised constitutive SA-pathway activation.

### Bacterial pathogen inoculation assays with *Pseudomonas syringae* pv. *tomato* DC3000

**Fig. 6:**
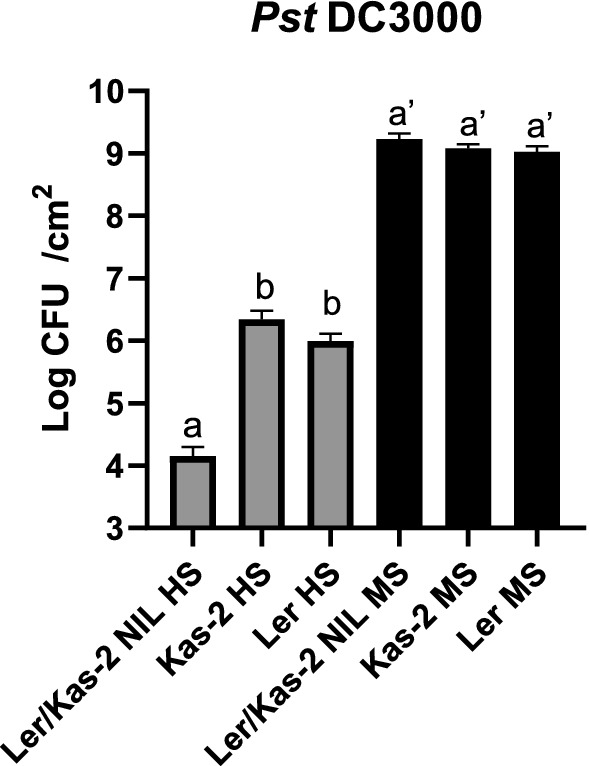
*Pseudomonas syringae* pv. *tomato* DC3000 (*Pst* DC3000) disease resistance phenotypes in 5-week-old L*er*/Kas-2 NIL, L*er* and Kas-2 plants grown at 14–16 °C. Bacterial numbers were determined at 72 h post-inoculation and expressed as colony forming units (CFU) per cm^2^ leaf area. Values are the mean from at least 12 biological replicates ± SD. Letters indicate values that are significantly different according to Tukey’s HSD test at *P* < 0.05

L*er*/Kas-2 NIL plants grown at 14–16 °C are more resistant to *Pseudomonas syringae* pv. tomato DC3000 (*Pst* DC3000) than L*er* or Kas-2 (Alcázar et al. 2010). To determine the effect of nutrition on bacterial disease resistance, *Pst* DC3000 was spray-inoculated in L*er*/Kas-2 NIL, L*er* and Kas-2 plants irrigated with HS or MS and grown at low temperature (14–16 °C) (Fig. 6). In the HS treatment, L*er*/Kas-2 NIL plants supported significantly less bacteria growth than L*er* or Kas-2 (Fig. 6). In contrast, no significant differences in *Pst* DC3000 growth were detected between L*er*/Kas-2 NIL and the parents in the MS treatment. The data were consistent with the suppression of L*er*/Kas-2 HI due to compromised constitutive activation of SA-pathway by MS treatment. We further observed that overall bacteria growth was significantly higher in plants irrigated with MS than HS, probably due to the higher nutrient availability in the apoplast of MS-irrigated plants.

### Analysis of hydrogen peroxide, nitric oxide, nitrate reductase, superoxide dismutase (SOD) and catalase (CAT) activities

**Fig. 7:**
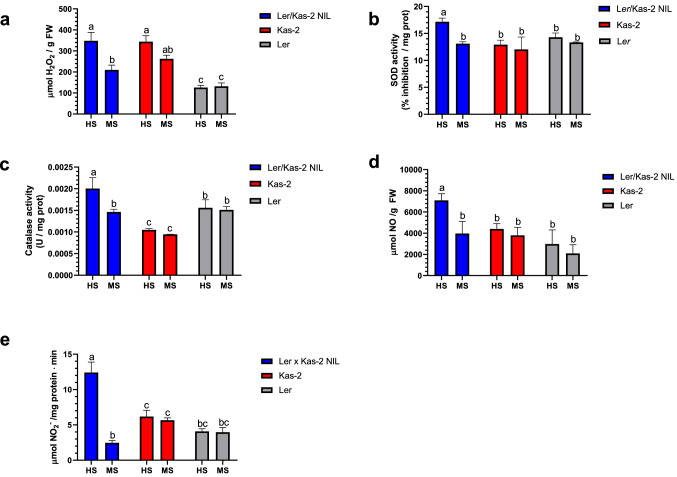
Quantitation of hydrogen peroxide (H_2_O_2_) levels **a**, SOD activity **b**, catalase activity **c**, nitric oxide (NO) levels **d** and nitrate reductase (NR) activity **e** in 5-week-old L*er*/Kas-2 NIL, L*er* and Kas-2 plants grown on soil at 14–16 °C and irrigated with HS or MS solutions. Values represent the average ± SD from ten biological replicates. Letters indicate values that are significantly different according to Tukey’s HSD test at *P* < 0.05

The generation of ROS and RNS is part of the plant defense response (Marino et al. 2012; Bellin et al. 2013; Wendehenne et al. 2014). To determine whether L*er*/Kas-2 HI associated with changes in ROS and/or RNS levels, we determined H_2_O_2_ and nitrite-derived NO levels, SOD, CAT, and NR activities in L*er*/Kas-2 NIL, L*er* and Kas-2 parents grown on soil at 14–16 °C and irrigated with HS or MS (Fig. 7).

L*er*/Kas-2 NIL irrigated with HS and showing incompatible phenotypes did not exhibit significant differences in H_2_O_2_ levels compared to the near isogenic Kas-2 parent under the same irrigation conditions (Fig. 7a). However, SOD and CAT activities were significantly higher in L*er*/Kas-2 NIL irrigated with HS (incompatible) compared to MS (compatible) and the parental lines Kas-2 or L*er*, irrigated with HS or MS (Fig. 7b, c). The data suggested that ROS produced in L*er*/Kas-2 NIL plants irrigated with HS was efficiently scavenged by enhanced CAT activity, leading to similar H_2_O_2_ levels to the Kas-2 near isogenic parent.

Conversely, L*er*/Kas-2 NIL irrigated with HS accumulated significantly higher NO than L*er*/Kas-2 NIL irrigated with MS or the parents irrigated with MS or HS (Fig. 7d). NR activity, which is a source of NO, was also significantly higher in L*er*/Kas-2 NIL irrigated with HS than MS, and L*er* or Kas-2 under either irrigation conditions (Fig. 7e). Therefore, autoimmune phenotypes in L*er*/Kas-2 correlated with higher NR activity and NO content. This might be due to the limited capacity of the L*er*/Kas-2 NIL to scavenge RNS.

### Contribution of ROS and NO to L*er*/Kas-2 hybrid incompatibility

**Fig. 8:**
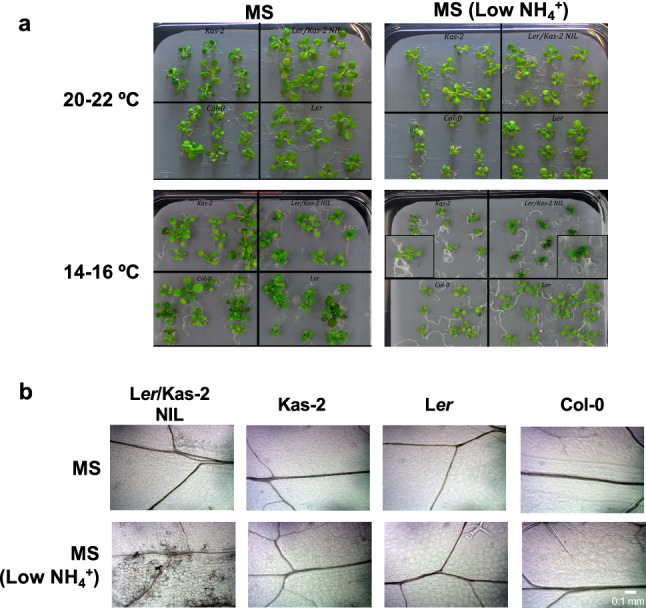
**a** Phenotype of 14-day-old L*er*/Kas-2 NIL, L*er*, Kas-2 and Col-0 plants grown in vitro on MS and low ammonium MS (1 mM NH_4_^+^) at 20–22 °C and 14–16 °C. **b** Dwarfism and cell death are reconstituted in L*er*/Kas-2 NIL plants grown at low temperature under low ammonium

**Fig. 9:**
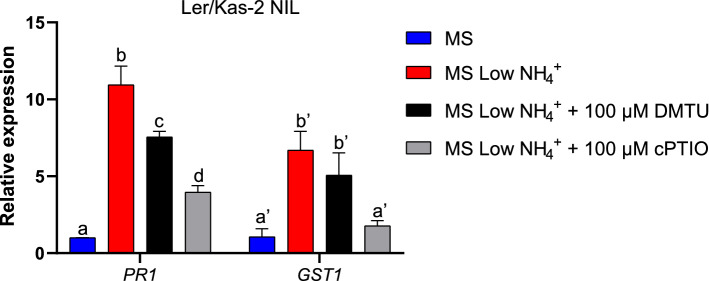
Quantitative gene expression analyses of *PR1* and *GST1* in L*er*/Kas-2 NIL plants grown in vitro on MS or low ammonium MS at 14–16 °C and treated with 100 µM DMTU (1,3-dimethyl-2-thiourea) or 100 µM cPTIO [2-(4-carboxyphenyl)-4,4,5,5-tetramethylimidazoline-1-oxyl-3-oxide]. qRT-PCR analyses were performed on at least three biological replicates with three technical replicates each. Letters indicate values that are significantly different according to Tukey’s HSD test at *P* < 0.05

In vitro growth of L*er*/Kas-2 NIL seedlings on low ammonium MS (MS containing 1 mM NH_4_^+^) reconstituted dwarfism and cell death at 14–16 °C (Fig. 8). To further investigate the contribution of NO and H_2_O_2_ to HI, the expression of *PR1* and *GST1* was determined in L*er*/Kas-2 NIL seedlings treated with the NO inhibitor cPTIO (2-(4-carboxyphenyl)-4,4,5,5-tetramethylimidazoline-1-oxyl-3-oxide) and the H_2_O_2_ scavenger 1,3-dimethyl-2-thiourea (DMTU). cPTIO and less markedly DMTU treatment, dampened the expression of *PR1* in L*er*/Kas-2 NIL plants grown on low ammonium MS (Fig. 9). In contrast, *GST1* expression was only significantly reduced by cPTIO treatment (Fig. 9). The data were consistent with a major contribution of NO to L*er*/Kas-2 immune-related HI, although ROS inhibition also partly suppressed hallmarks of constitutive SA-pathway activation.

### Dependence of L*er*/Kas-2 hybrid incompatibility on NPR1

**Fig. 10:**
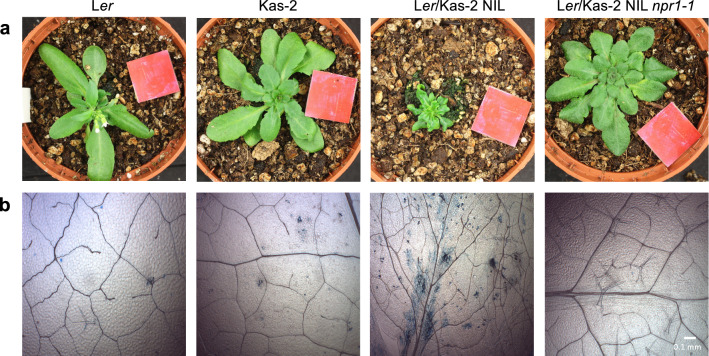
**a** Phenotype of 5-week-old L*er*/Kas-2 NIL *npr1-1*, L*er*/Kas-2 NIL, L*er* and Kas-2 plants grown on soil at 14–16 °C. **b** Microscope visualization of cell death by trypan blue staining

Given the contribution of NO to NPR1 signaling (Tada et al. 2008), and the dependence of L*er*/Kas-2 HI on SA-pathway (Alcázar et al. 2009), we investigated the requirement of NPR1 to the establishment of L*er*/Kas-2 HI. The L*er*/Kas-2 NIL was crossed to *npr1-1* (Col-0) mutant to isolate plants carrying the fixed *npr1-1* mutation in combination with homozygous L*er* and Kas-2 incompatible loci on QTL3 (*RPP1*-like L*er*), QTL4 (*SRF3* Kas-2) and QTL5 (Kas-2) (Alcázar et al. 2009, 2010). Compared to L*er*/Kas-2 NIL, quadruple homozygous L*er*/Kas-2 *npr1-1* plants did not show dwarfism and cell death at low temperature (14–16 °C), which are hallmarks of L*er*/Kas-2 HI. We concluded that NPR1 is required for the full establishment of L*er*/Kas-2 HI (Fig. 10).

## Discussion

Nitrogen is taken up by roots in form of nitrate or ammonium. In *Arabidopsis*, there are more than 10 nitrate transporters (NRT) which are tissue-specific and show low or high affinity toward nitrate (Masclaux-Daubresse et al. 2010; Wang et al. 2012; Noguero and Lacombe 2016). Nitrate is then translocated to the shoot by xylem vessels or assimilated in root cells. Nitrate can be reduced into nitrite (NO_2_^−^) by cytosolic NR that uses NAD(P)H as electron donor and generates NO, a multitasked signaling molecule (Domingos et al. 2015). NR activity is temperature-dependent and negatively feedback-regulated by nitrite levels (Cheeseman and Tankou 2005; Sanz-Luque et al. 2015). Nitrite is converted into ammonium by plastidial NITRITE REDUCTASE (NiR) and finally assimilated into amino acids (Stitt 1999). Ammonium is fixed into amino acids via the glutamine synthetase/glutamate synthase (GS/GOGAT) cycle coupled to the enzymatic activities of several aminotransferases, such as asparagine synthetase and glutamine synthetase (Coruzzi 2003). Glutamine (Gln) and, in less quantity asparagine (Asn), are transported to the shoot by the plant vascular system for their use in anabolic processes (Tischner 2000). Ammonium is a fast intake source for nitrogen. The levels of ammonium are highly variable due to leaching, nitrification and denitrification, presence of fertilizers among other factors (Britto and Kronzucker 2002). Massive farming and fertilization often lead to high ammonium concentration in soils. Even though nitrogen input increases crop productivity, it also increases nitrogen resources for pathogenic growth (Solomon et al. 2003). As such, disease development might be compromised or favored under different nitrogen fertilization regimes (Mur et al. 2017).

Wang et al. (2013) reported the regulation of *snc1-1* (*suppressor of npr1-1, constitutive 1*), *cpr1* (*constitutive expresser of PR genes 1*) and *nudt6-2* (*nudix hydrolase homolog 6–2*) *nudt7* autoimmune phenotypes by the ratio between ammonium and nitrate. The proposed underlying mechanism involves the differential modulation of NO production, which may act as EDS1 (ENHANCED DISEASE SUSCEPTIBILITY 1) signaling amplifier. Low NH_4_^+^/NO_3_^−^ ratio stimulates *snc1* autoimmunity, whereas high NH_4_^+^/NO_3_^−^ ratio suppresses autoimmunity including dwarfism and cell death (Wang et al. 2013). The hypersensitive response (HR) triggered by *P. syringae* pv. *phaseolicola* was also exacerbated in tobacco plants fed with nitrate compared to ammonium, and this also associated with enhanced NO generated by NR activity, and higher SA levels (Gupta et al. 2013). Furthermore, ammonium treatment elevated the levels of total amino acids and sugars in the apoplast, thus supporting higher bacteria growth (Gupta et al. 2013). Ammonium leads to lower levels of NO by suppression of NR activity, which might support the effect of nutrition on NO production (Planchet et al. 2005; Gupta et al. 2013). In agreement with previous works, we find that high ammonium suppresses L*er*/Kas-2 immune-related HI and this associates with lower NO production and NR activity (Figs. 3, 7b, c). In addition, L*er*/Kas-2 HI is also EDS1 and SA-pathway dependent thus supporting the interaction between NO and EDS1/SA pathways (Durner et al. 1997; Alcázar et al. 2009). Ammonium feeding also supported enhanced bacteria growth in *Arabidopsis* plants, probably due to higher nutrient availability (Gupta et al. 2013). However, higher nitrate levels did not exacerbate autoimmunity in L*er*/Kas-2 NIL (Fig. 4), which otherwise accumulated constitutively higher NO levels at low temperature (Fig. 7b).

NO participates in different aspects of plant defense, such as the regulation of gene expression during HR and accelerates the kinetics of HR formation (Durner et al. 1997; Delledonne et al. 2001; Bellin et al. 2013; Mur et al. 2017). NO is also required for the nitrosylation of TGA-class transcription factors required for SA-dependent gene expression (Lindermayr and Durner 2009; Lindermayr et al. 2010; Yu et al. 2014). Furthermore, NO is used as substrate for posttranslational modification of NPR1 by S-nitrosylation (Lindermayr et al. 2010)*.* The NPR1 protein oligomerizes in the cytoplasm and upon S-nitrosylation, a conformational change is induced that delivers NPR1 monomers to the nucleus, leading to gene-expression changes (Mou et al. 2003; Tada et al. 2008; Lindermayr et al. 2010). In the SA–NO interaction, SA has been reported to stimulate NO synthesis (Zottini et al. 2007) acting synergistically upstream of NPR1 (El-Shetehy et al. 2015)*.* Interestingly, the L*er/*Kas-2 HI is also found to be NPR1-dependent, thus suggesting the participation of the NO/SA/NPR1 module in the expression of L*er*/Kas-2 HI.

In addition to RNS, the production of ROS is a characteristic of defense activation. PAMP recognition by immune receptors stimulates plasma-membrane NADPH oxidases to produce apoplastic ROS (Torres et al. 2002). In addition, ROS triggers defense-related gene expression changes, stimulates HR, SA and NO synthesis (Torres and Dangl 2005; Suzuki et al. 2011). Indeed, the interaction between NO and ROS seems to be important for the generation of HR (Delledonne et al. 2001; Torres et al. 2002; Yun et al. 2011). Despite we did not detect significant differences in the levels of H_2_O_2_ between L*er*/Kas-2 NIL plants and the near isogenic Kas-2 parent irrigated with HS or MS at 14–16 °C (Fig. 7a), DMTU (1,3-dimethyl-2-thiourea) treatment partly attenuated the constitutive overexpression of *PR1* in L*er*/Kas-2 NIL plants grown on low ammonium MS (Fig. 9). The data suggest a major contribution of NO to the occurrence of L*er*/Kas-2 HI. However, the participation of ROS to L*er*/Kas-2 incompatibility cannot be fully excluded based on gene expression analyses.

Overall, we provide evidence for the suppressive effect of high ammonium in the occurrence of immune-related hybrid incompatibilities in *Arabidopsis*, and the major contribution of NO and NPR1 signaling on L*er*/Kas-2 HI.

### *Author contribution statement*

KEA and RA: conceived and designed research; KEA and LCD-N: conducted the experiments; KEA and RA: analyzed the data; KEA and RA: wrote the manuscript. All authors read and approved the manuscript.

## Supplementary Information

Below is the link to the electronic supplementary material.

Supplementary file1 (XLSX 10 KB)

## Data Availability

All data generated or analyzed during this study are included in this published article and its supplementary information files.

## Abbreviations

HI: Hybrid incompatibility
HR: Hypersensitive response
HS: Hoagland’s nutrient solution
MS: Murashige and Skoog basal medium
NIL: Near isogenic line
NPR1: NONEXPRESSER OF PR GENES 1
NR: Nitrate reductase
NO: Nitric oxide
RNS: Reactive Nitrogen Species
ROS: Reactive Oxygen Species
SA: Salicylic acid
